# The Corticospinal Tract: A Biomarker to Categorize Upper Limb Functional Potential in Unilateral Cerebral Palsy

**DOI:** 10.3389/fped.2015.00112

**Published:** 2016-01-06

**Authors:** Ellen Jaspers, Winston D. Byblow, Hilde Feys, Nicole Wenderoth

**Affiliations:** ^1^Neural Control of Movement Laboratory, Department of Health Sciences and Technology, ETH Zurich, Zurich, Switzerland; ^2^Movement Neuroscience Laboratory, Department of Sport and Exercise Science, University of Auckland, Auckland, New Zealand; ^3^KU Leuven, Department of Rehabilitation Sciences, Research Group of Neuromotor Rehabilitation, Leuven, Belgium; ^4^KU Leuven, Department of Kinesiology, Movement Control and Neuroplasticity Research Group, Leuven, Belgium

**Keywords:** cerebral palsy, upper limb, corticospinal tract, reorganization, biomarker, categorization

## Abstract

Children with unilateral cerebral palsy (CP) typically present with largely divergent upper limb sensorimotor deficits and individual differences in response to upper limb rehabilitation. This review summarizes how early brain damage can cause dramatic deviations from the normal anatomy of sensory and motor tracts, resulting in unique “wiring patterns” of the sensorimotor system in CP. Based on the existing literature, we suggest that corticospinal tract (CST) anatomy and integrity constrains sensorimotor function of the upper limb and potentially also the response to treatment. However, it is not possible to infer CST (re)organization from clinical presentation alone and conventional biomarkers, such as time of insult, location, and lesion extent seem to have limited clinical utility. Here, we propose a theoretical framework based on a detailed examination of the motor system using behavioral, neurophysiological, and magnetic resonance imaging measures, akin to those used to predict potential for upper limb recovery of adults after stroke. This theoretical framework might prove useful because it provides testable hypotheses for future research with the goal to develop and validate a clinical assessment flowchart to categorize children with unilateral CP.

## General Introduction

With a prevalence of 1 in 500 newborns, cerebral palsy (CP) is the leading cause of childhood physical disability (1). This review focuses on upper limb function in children with unilateral CP, which accounts for 38% of the CP group (2). These children typically present with delays in sensorimotor development and in the acquisition of gross and fine motor upper limb skills. Irrespective of the severity of the brain lesion, they experience lifelong disabilities that put a high emotional and financial burden on families, caretakers, and society (3, 4). To maximize the child’s functional potential and subsequent independence in life, adequate treatment planning is essential. However, treatment optimization is challenged by the large heterogeneity in the clinical presentation of children with unilateral CP. Despite the rapid increase of evidence-based therapy management, large variability in treatment response persists; Novak et al. (5) recently showed that 70% of the available interventions for children with CP have highly variable efficacy and the existing clinical assessments and outcome measurements fail to accurately predict treatment response (5). A stratified therapy approach could further optimize treatment planning, thereby increasing the odds that a child reaches its maximal functional potential within the constraints imposed by the structural damage of the brain. The strategic and economic significance of the identification of subgroups or strata of patients based on clinical biomarkers has been clearly demonstrated in other areas, such as oncology (6, 7). Exploring the clinical merit of such an approach in CP seems warranted.

The first step toward stratification is the identification of clinically relevant biomarkers. Literature has indicated that the clinical assessment of sensorimotor function alone is not enough but may be complemented with information about neural, structural and functional integrity. The heterogeneous nature of brain lesions underlying CP might constitute a crucial factor in explaining treatment response variability. Brain lesions range from relatively localized damage to the motor pathways (often seen when lesions occur after 24 weeks of gestation), to severe malformations typically seen when the incident occurs during the first months of pregnancy. Structural MRI has been used to derive neural biomarkers such as lesion location and extent, often combined with the time-point of the insult (8). However, gross anatomy or timing of the lesion remains only a moderate predictor of a child’s sensorimotor function (9, 10), even when applied at the group level. This raises the question why simple markers describing lesion anatomy are relatively uninformative in children with CP. Part of the answer is that the brain is still highly plastic in the early stages of development, undergoing vast time-dependent maturational changes, making specific parts of the brain particularly vulnerable to injury (11). Following injury at this phase of development, plasticity permits alterations from the pre-programmed pathway of brain organization (12, 13). As a result, the final “wiring” of the sensorimotor system might deviate from that expected, a phenomenon that is unique to unilateral CP. If the pattern and extent of “re-wiring” can be identified, it may offer clinical utility.

In the first part of the review, we provide a concise overview of typical and disrupted neural development of the human brain and summarize the available knowledge related to how brain damage impacts on sensorimotor function in unilateral CP. In the second part of this review, we propose the working hypothesis that the initial brain damage and concurrent structural reorganization of the sensorimotor system (and most notably the corticospinal tract, CST) form a primary source of variability among children with unilateral CP, and constrain the maximal functional potential a child can theoretically reach. Since two children can present with similar sensorimotor function yet differ largely regarding the underlying anatomical substrate, we propose a systematic evaluation of the CST to infer the wiring pattern at the level of the individual child with unilateral CP. With this review, we intend to generate testable hypotheses to identify biomarkers that go beyond the traditional clinical assessments and that allow categorizing children based on their CST wiring pattern. Such categorization might prove useful in a clinical context and in the long run, these insights will further advance research in the field of therapy stratification in unilateral CP.

## Anatomical Pathways for Upper Limb Movements

Voluntary upper limb movements originate primarily from the contralateral motor cortex, which receives input from frontal and parietal areas that play an important role in higher-order sensorimotor processing. The motor cortex is divided into the primary motor cortex (M1), premotor cortex (PM), cingulate motor area (CMA), and supplementary motor area (SMA) (14). These areas are densely interconnected within one hemisphere via association tracts, and connect with homologous areas of the opposite hemisphere via commissural tracts. The CST constitutes the major motor output pathway. It is formed by large pyramidal neurons from M1, which converge with fibers from SMA, PM, the somatosensory cortex, and the posterior parietal cortex. The CST passes through the corona radiate, the posterior limb of the internal capsule, and the cerebral peduncles, and crosses at the level of the pyramidal decussation into the lateral spinal cord. A small portion (10%) of the CST also descends anteriorly into the ipsilateral spinal cord. These uncrossed anterior projections are thought to primarily innervate proximal and axial muscles, rather than distal forearm and hand muscles (15, 16). However, the exact functional role of the uncrossed anterior projections remains unclear (17, 18).

**Figure 1:**
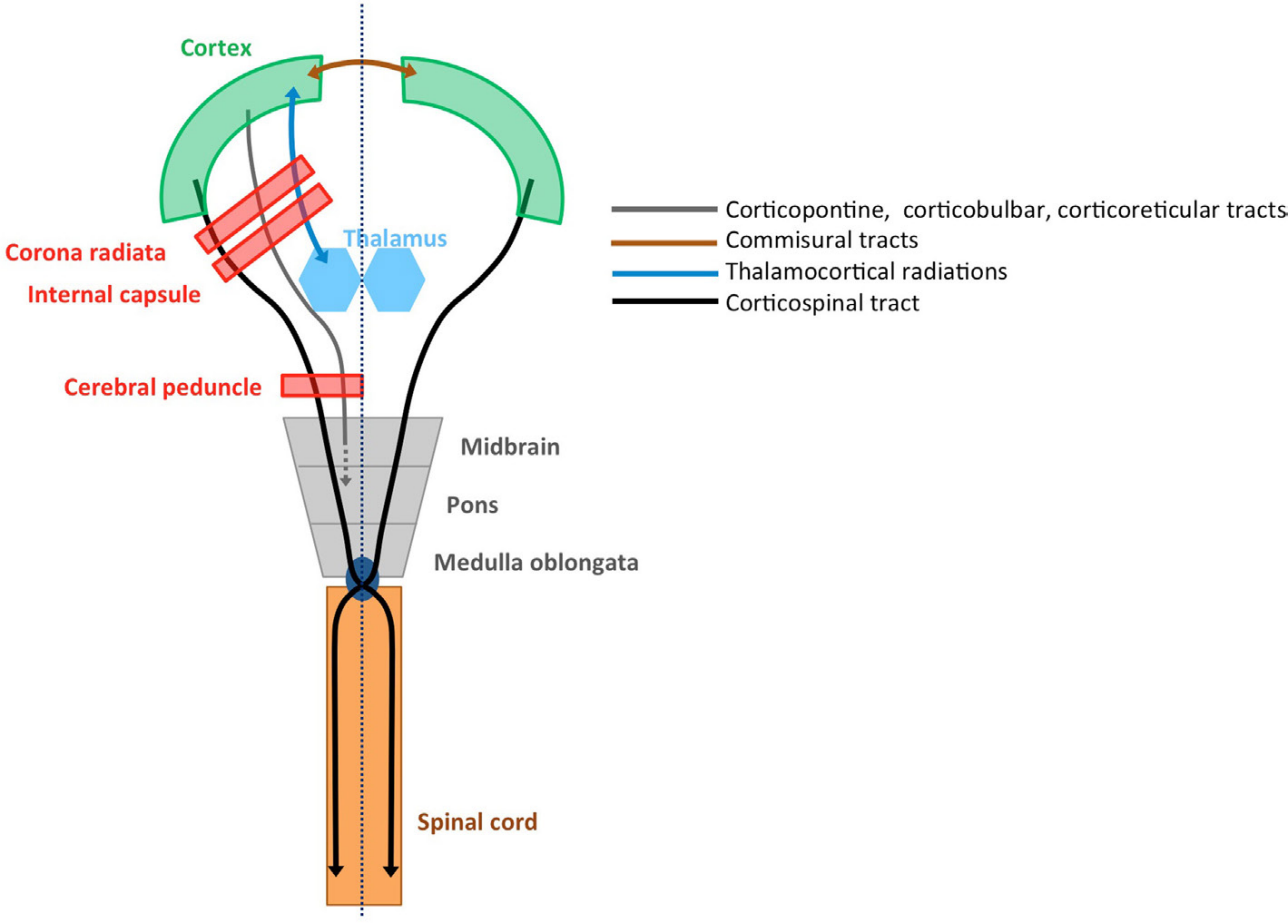
**Structural connectivity of the sensorimotor system**. Schematic overview of relevant tracts and structures (adopted from Ref. 20).

The afferent cortical input needed for the accurate execution of movements, i.e., the sensory information, is ensured via the thalamocortical radiations into the motor areas and the primary and secondary sensory areas (19). An overview of relevant tracts and structures related to upper limb sensorimotor function is provided in Figure 1.

## Lesion Types and “Re-Wiring” of Major Tracts Following Early Brain Damage

### Malformations (Week 0–24)

**Figure 2:**
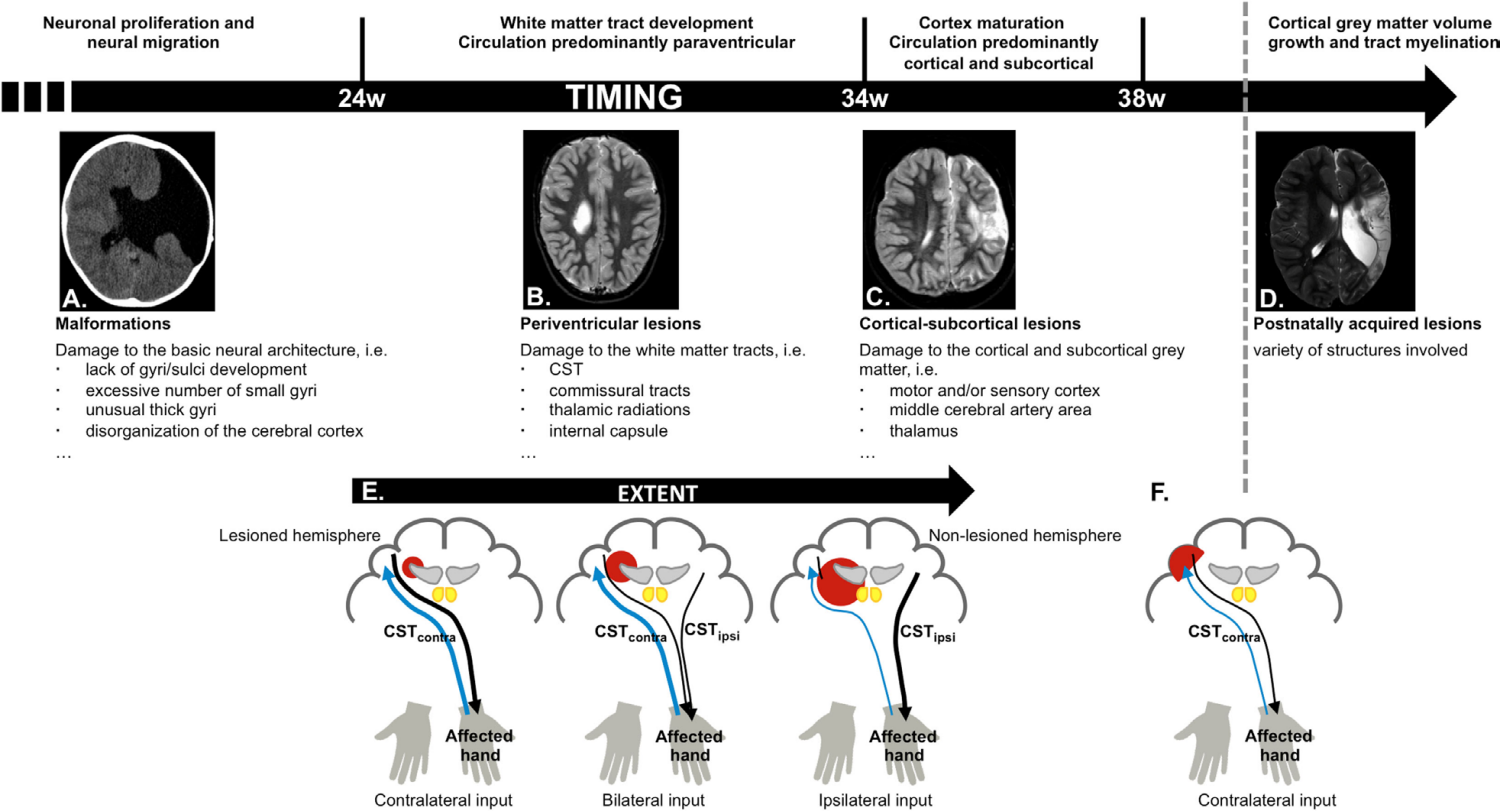
**Schematic overview of cerebral development and structures more vulnerable to damage depending on the stage of brain maturation and possible reorganization of the motor and sensory system, based on time-point of occurrence and lesion extent**. **(A)** Malformations, caused by an insult in the first 24 weeks of gestation (schizencephaly); **(B)** periventricular lesions, which typically occur around week 24–34 of gestation, and affect the corticospinal tract (CST); **(C)** cortical–subcortical lesions, which typically occur after 34 weeks of gestation and primarily affect the motor and/or sensory cortex; **(D)** postnatally acquired lesions, which occur after 28 days after birth until age 2 years; **(E)** different types of motor reorganization are typically seen following periventricular lesions (CST, black), whereby the reorganization pattern depends on the extent of the lesion. The general pattern of the sensory pathways is preserved (thalamocortical radiations, blue); **(F)** crossed CST projections from the lesioned hemisphere are at least partially intact following cortical–subcortical or postnatally acquired lesions.

In the first 24 weeks of gestation the brain undergoes major morphological changes, such as the formation of the cerebral hemispheres, the folding of the cortex, and the shaping of the ventricular system (11). Lesions occurring before week 24 therefore typically result in malformations (Figure 2A), such as a lack of gyri or sulci development or an excessive number of small gyri, unusually thick convolutions, or a disorganization of the cerebral cortex (e.g., schizencephaly). These lesions occur in <10% of the children with unilateral CP (8, 9).

### Periventricular White Matter Lesions (Week 24–34)

During week 24–34, brain maturation is predominantly characterized by white matter tract development. Association tracts (cortical–cortical connections) and afferent/efferent projection tracts (connecting the cortex with subcortical nuclei, the cerebellum, and the spinal cord) arise from the neurepithelium surrounding the lateral ventricles. Importantly, each hemisphere initially develops bilateral crossed and uncrossed descending efferent projections that form the CST. During typical development, the ipsilateral uncrossed projections (CST_ipsi_) gradually weaken and the contralateral crossed projections (CST_contra_) strengthen. This process, known as competitive withdrawal, occurs at the termination point of corticospinal neurons within the spinal cord (21), resulting in predominantly contralateral control of the upper limb.

White matter tract development is accompanied by a local increase in blood flow around the lateral ventricles. Hence, insults occurring between week 24 and 34 most frequently result in periventricular white matter lesions (Figure 2B), which account for around half of the children with unilateral CP (8, 9). The CST has already reached the cervical cord by 24 weeks of gestation (22), and lesions that occur during this period frequently damage the CST and the internal capsule (23), resulting in reduced integrity of the motor tract and the posterior/reticular limb of the internal capsule (24), for review see Ref. (25). These lesions also compromise the typical competitive withdrawal process of the CST (26), thereby causing a unique “re-wiring” within the sensorimotor system in unilateral CP: the existing uncrossed projections from the non-lesioned hemisphere (CST_ipsi_) gain control of the affected hand (13), and are strengthened during further development and environmental interactions. Conversely, the weaker crossed projections (CST_contra_) from the lesioned hemisphere withdraw, at least partly (12, 26). Eventually, the non-lesioned hemisphere can become equipped with fast-conducting uncrossed projections to the affected upper limb (21, 23, 27). Importantly, this “re-wiring” pattern is influenced by lesion extent, whereby only larger lesions seem to cause a “shift” of the CST toward the non-lesioned hemisphere (23, 27) (Figure 2E). However, the functional relevance of ipsilateral control of the affected hand in children with unilateral CP compared to typically developing children remains ambiguous, as there are currently no known associations between neurophysiological lateralization indices and upper limb function.

Cortical synapses of the afferent thalamocortical radiations are formed later than the CST (28), such that afferent tracts are much less affected than efferent tracts. Nonetheless, reduced integrity of the posterior thalamocortical radiations has been reported in children with periventricular lesions (29, 30). Thalamocortical radiations also follow a different pattern of reorganization, whereby the sensory afferents seem to bypass even larger lesions to reach the contralateral cortex (13, 31, 32) (Figure 2E). Although the general wiring pattern is preserved for the afferent pathways, and sensory input of each hand is connected to the contralateral cortex, there might be profound reorganization within the primary sensory cortex of the lesioned hemisphere (27).

### Cortical-Subcortical Lesions (Week 34–38, and Up To 28 Days After Birth)

Week 34–38 of gestation is characterized by further maturation of the tracts (synaptic production and myelination) (33), causing a vast improvement of fetal movement quality, alertness, and visual function (34). This maturation coincides with a migration of the area of blood flow toward the cortical and subcortical areas. Consequently, lesions occurring after 34 weeks of gestation or around birth typically affect cortical or subcortical gray matter of the central and parasagittal areas (Figure 2C) (8, 35). These cortical–subcortical “infarct-like” lesions occur in 20–30% of the children with unilateral CP (8, 9) and often do not extend so far medially as to also affect the periventricular white matter (26). As a consequence, crossed CST projections from the lesioned hemisphere are usually at least partially intact and “re-wiring” to the non-lesioned sensorimotor areas is less frequently seen (26, 31, 32) (Figure 2F).

### Postnatally Acquired Lesions (Up To Age 2 years)

The first 2 years of life are a highly dynamic period and perhaps the most critical phase of postnatal brain development, characterized by structural brain growth and a rapid development of a whole range of cognitive and motor functions (36). Lesions occurring between 28 days after birth and before the age of 2 years are categorized as postnatally acquired and represent around 15% of the lesions in children with unilateral CP (9). Postnatally acquired lesions entail a variety of affected structures, whereby cortical damage in the area of the cerebral medial artery and deep gray matter structures is most prevalent (Figure 2D) (9). Sensory reorganization toward the non-lesioned hemisphere has not yet been described in these children (31) (Figure 2F).

## Early Brain Damage and Upper Limb Deficits

At a general level, the severity of upper limb sensorimotor deficits in unilateral CP depends on the time of the insult, as well as on the location and extent of the lesion (8, 35, 37, 38). For example, periventricular lesions that occur between week 24 and 34 on average lead to fewer motor and tactile deficits and better arm and hand function than cortical–subcortical lesions that occurred after 34 weeks or around birth, or than postnatally acquired lesions (9, 26, 31, 39). However, if the early lesion is large and causes substantial “re-wiring” such that the affected upper limb receives input from the non-lesioned ipsilateral hemisphere, this results in poorer performance compared to children with periventricular lesions with contralateral control of the affected upper limb (26, 31). Additionally, the structural integrity of the CST and thalamocortical radiations might further modulate the extent of upper limb deficits (40, 41). Lastly, basal ganglia/thalamus damage often results in poor upper limb sensorimotor function, reach and grasp abilities, and bimanual hand use, irrespective of the timing of the brain lesion and potential reorganization (9, 10, 39, 42).

**Figure 3:**
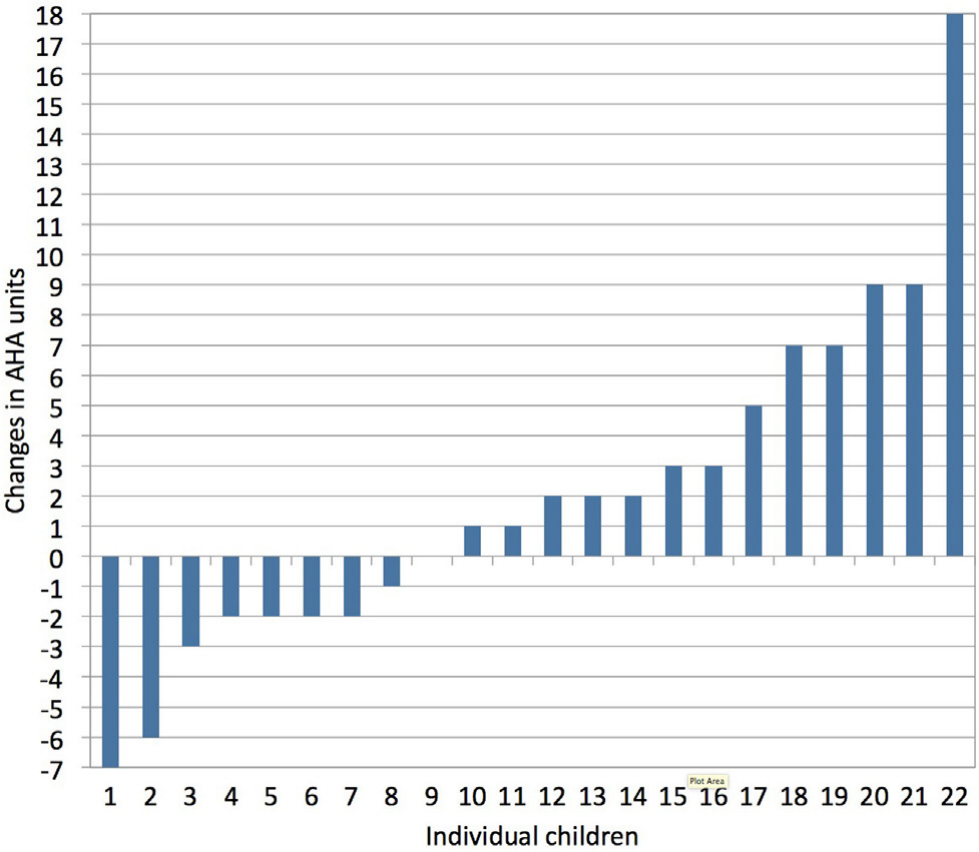
**Individual treatment responses following a constraint-induced movement therapy (CIMT) program, as measured with the assisting hand assessment (AHA, bimanual hand use)**. Vast inter-individual variation in treatment response is seen following CIMT (1 h/day, 5 days/week for a period of 10 weeks in children age 4–12 years). Changes in AHA score of 5 or more units surpass the smallest detectable difference (personal communication with Klingels based on study results published in Ref. 46).

Upper limb motor deficits typically include muscle weakness, spasticity, dystonia, and muscle shortening (43). More than 75% of children with unilateral CP also experience deficits in exteroception, proprioception, two-point discrimination, and/or stereognosis (44). Together, these sensorimotor deficits compromise the acquisition of gross and fine motor skills, resulting in less (effective) use of the affected hand in unimanual and bimanual activities (43). Adequate treatment selection and planning are important to maximize a child’s upper limb functional abilities. However, there is a lack of strong evidence in favor of any particular upper limb therapy approach in children with unilateral CP (45). This is likely due to the heterogeneous nature of lesions in these children and the highly variable treatment response at the level of the individual child compared to group averages (Klingels, personal communication), as illustrated in Figure 3.

Together, these results have led to a general consensus within the CP community that biomarker-based treatment planning offers a new opportunity to further advance upper limb functional outcome. However, it remains unclear what biomarkers are clinically relevant and how they can be combined to guide therapy decisions. Recent research has suggested that neural biomarkers related to the specific wiring pattern of the CST may become predictive for treatment outcome (47, 48). This supports our working hypothesis that the initial brain damage and concurrent structural reorganization of the sensorimotor system form a major source of variability among children with unilateral CP. In the next paragraph, we propose an approach of how to infer the CST wiring pattern and connectivity strength at the level of the individual child with unilateral CP.

## Probing the CST

The CST wiring pattern is usually not immediately apparent from how the child presents clinically, and children with unilateral CP might have similar upper limb sensorimotor deficits, despite a different underlying CST wiring pattern. Additionally, CST wiring might also aid in further explaining the heterogeneity in upper limb outcome within the group of children with unilateral CP. Two questions might be of relevance for clinical decision-making: first, does the affected hand receive significant input from the non-lesioned hemisphere via uncrossed CST_ipsi_ fibers or from the lesioned hemisphere (CST_contra_)? Second, can further important information be derived from estimating the “connectivity strength” of the CST in the lesioned hemisphere, i.e., its quality or structural integrity?

Here, we present a theoretical framework, integrating behavioral, neurophysiological, and medical imaging measures to allow a systematic evaluation of CST wiring and connectivity strength. The proposed flowchart is purely hypothetical at this point but provides a series of testable ideas on how the individual CST wiring pattern of a child with unilateral CP can be inferred from different measurements. It is important to note, however, that the development of a clinically applicable assessment flowchart requires direct validation in children with unilateral CP, which will be the focus of future research.

**Figure 4:**
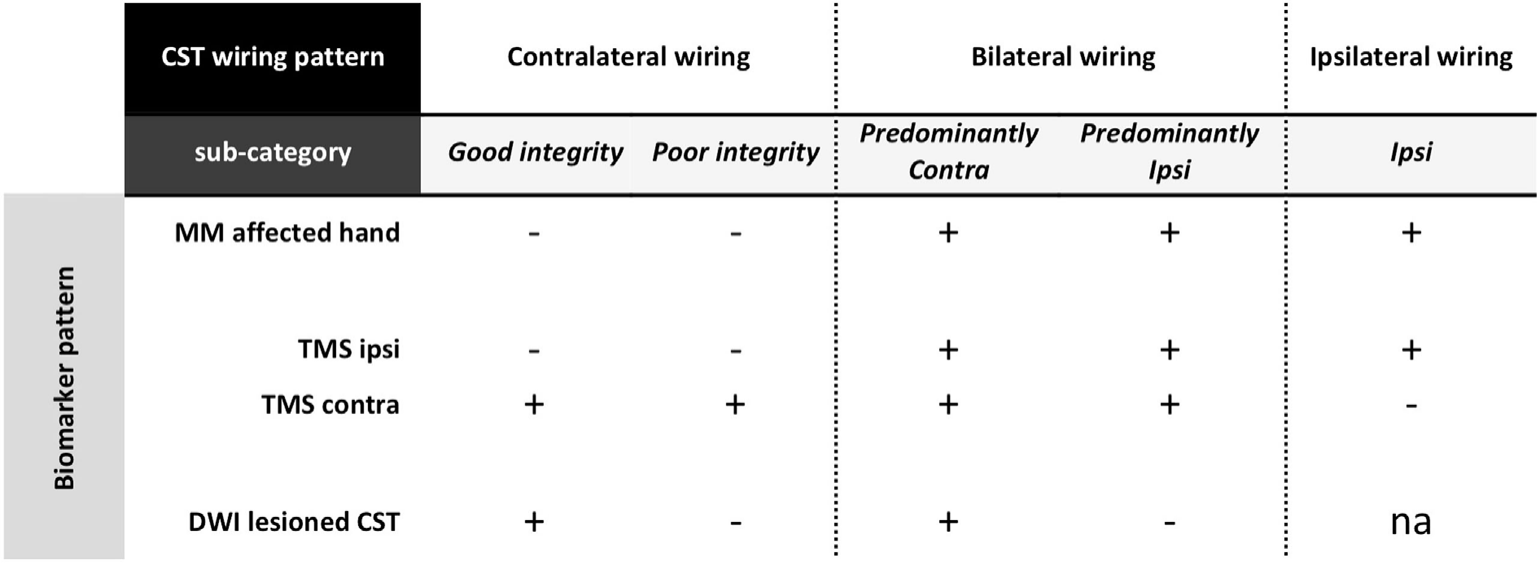
**Categorization based on a stepwise evaluation of CST wiring pattern and structural integrity in unilateral CP**. First, if MM are present at the affected hand, this suggests some ipsilateral control of the affected hand. Further investigation with TMS will help to identify whether children have a bilateral or a unique unilateral control of the affected hand. Children with a pure ipsilateral wiring pattern form a first category in this scheme. For those children with bilateral control of the affected hand, DWI allows to determine the structural integrity of the lesioned CST_contra_. Here, a further categorization entails those with good structural integrity vs. poor structural integrity (i.e., affected hand predominantly controlled via the lesioned CST_contra_ or via the non-lesioned CST_ipsi_, respectively). In children with unilateral CP who do not present with MM in the affected hand, TMS will confirm the contralateral control via the lesioned CTS_contra_. Here, DWI can again be used to further categorize children into those with good vs. poor structural integrity of the lesioned CST_contra_. CST, corticospinal tract; MM, mirror movements; TMS, transcranial magnetic stimulation; DWI, diffusion-weighted imaging; ipsi, ipsilateral hemisphere with respect to the affected hand; contra, contralateral hemisphere with respect to the affected hand; na, not applicable.

A simple behavioral measure to probe CST wiring could be the occurrence of *mirror movements (MM)*. MM refer to involuntary movements of one hand that mirror the intentional movement of the contralateral hand (49, 50). MM are part of the physiological motor pattern in typically developing children up to age 10 years and increase with increasing task complexity (51). The relationship between age and the occurrence of MM is less straightforward in unilateral CP, i.e., MM are driven by different phenomena in the affected and non-affected hand (52). Moreover, it also appears that highly repetitive and simple motor tasks are more appropriate to assess the occurrence of pathological mirror movements (52). *MM in the affected hand* (i.e., mirroring intentional movements of the non-affected hand) have been proposed to be indicative for one motor cortex controlling both hands, i.e., ipsilateral or bilateral “re-wiring” of the CST (26). MM in the non-affected hand seem more related to sensorimotor impairment of the affected hand rather than to the CST wiring pattern (50, 53). We propose the assessment of MM in the affected hand as a non-invasive, low-risk clinical biomarker to probe CST wiring and categorize children with unilateral CP (Figure 4). However, adequate interpretation of the frequency and magnitude of MM would benefit from further standardization of the assessment in terms of task complexity (51), but also through the use of a quantitative method based on, e.g., grip force measurements.

The sensorimotor system can also be assessed using *single-pulse TMS* over the hand area of the motor cortex in the lesioned and non-lesioned hemisphere to elicit MEPs in the affected hand (54). Absence of a descending CST projection from one hemisphere is assumed when even high stimulation intensities fail to elicit early MEP responses in the affected hand (23). Based on this neurophysiological biomarker, children with unilateral CP can be further categorized based on whether MEPs in the affected hand are elicited from the lesioned hemisphere only (contralateral wiring), from the non-lesioned hemisphere only (pure ipsilateral wiring), or from both hemispheres (bilateral wiring), as illustrated in Figure 4. Single-pulse TMS has been shown to be safe and well tolerated in children, i.e., the occurrence of seizures has not been reported, despite the growing variety of childhood neurological conditions being studied (55, 56 and for review see Ref. 57). However, while single-pulse TMS is put forward as a viable technology to increase our understanding of disorders of the developing brain, the application of this technique requires specialized training and should only be delivered by expert assessors within a child-friendly environment. One must note that children under age 10 years have a higher (resting and active) motor threshold compared to adults, which only decreases to adult levels by mid-adolescence (56, 58). Although eliciting an MEP in a relaxed muscle in children younger than 6 years might not be possible, even at maximum stimulator output (59), MEPs can certainly be elicited when the target muscle is active (21). On the other hand, TMS might be most informative in older children and certainly biomarker research might benefit most from focusing on older children and adolescents. In children with unilateral CP, the CST is not expected to reorganize after age 2 years (21), and thus information obtained from older children is still expected to generalize to the larger group of children with unilateral CP.

Lastly, in case of contralateral or bilateral “re-wiring,” we hypothesize that the structural integrity of the lesioned CST_contra_ will provide further insights that might aid clinical decisions. S*tructural imaging* techniques, particularly diffusion MRI and fiber tracking, might be promising imaging biomarkers to assess the contralateral input to the control of the affected hand. This will allow quantifying CST asymmetry between the lesioned and the non-lesioned hemisphere, using measures of fractional anisotropy or fiber count either at the level of the posterior limb of the internal capsule or cerebral peduncles (for review see Ref. 25). Increased asymmetry between both hemispheres has been related to more severe upper limb deficits in unilateral CP (good vs. poor contralateral wiring) and might help distinguishing predominant ipsilateral vs. predominant contralateral control of the affected hand in those children with a bilateral wiring pattern (Figure 4). While diffusion imaging provides detailed anatomical information that cannot be accessed with any other currently available imaging method, this technique also implies specific expertise to ensure an adequate and proper interpretation of the diffusion images (for review see Ref. 60). Moreover, given the length and noisiness of diffusion imaging acquisition protocols, it might not always be feasible in younger children. Future research will have to clarify whether or not the implementation of any diffusion-weighted imaging protocol in clinical practice is truly beneficial.

At present, it is speculative whether the biomarkers proposed here can be used to infer the underlying CST “(re-)wiring” pattern and structural integrity for individual children with unilateral CP. However, the utility of several of the suggested biomarkers has been demonstrated previously (see next paragraph). The proposed theoretical scheme generates five categories and concurrent hypotheses that can be tested empirically in future studies. Note, however, that the scheme as depicted here is only an example of what such an assessment flowchart might look like and awaits validation in children with unilateral CP. In the long run, this might pave the road for future studies investigating whether treatment allocation based on biomarkers characterizing the neuroanatomy of the individual child’s CST is feasible and advantageous compared to traditional approaches (see also Ref. 48).

## Experimental Evidence Supporting the Categorization Based on the CST

The five categories described in Figure 4 are based on knowledge about typical brain development, combined with empirical data from children with unilateral CP (23, 26, 31, 61). Here, we evaluate this categorization against existing evidence, based on clinical, (neuro)physiological, or medical imaging parameters. Any future flowchart developed on the basis of the presented hypothesis and concurrent new clinical evidence could benefit from the theoretical framework proposed here. However, the current depiction is only an example of what such an assessment flowchart might look like.

The first premise of our categorization relates to the wiring pattern of the CST, i.e., is the affected hand mainly controlled via the contralateral lesioned hemisphere or via the ipsilateral non-lesioned hemisphere? While the occurrence of ipsilateral control of the affected hand has not yet been systematically assessed, it is estimated to occur in 30–60% of the children with unilateral CP (23, 62, 63), and many children present with a mixed response pattern (23, 31, 63). Ipsilateral “re-wiring” ensures the development of (some) upper limb skills despite severe CST damage (23, 26), though it is an insufficient substitute for the typical contralateral control (17) and (near) normal hand function is only seen when the affected hand is controlled via the CST_contra_ of the lesioned hemisphere (23, 26, 31). No or minimal MM of the affected hand (23), as well as the absence of ipsilateral control of the affected hand based on TMS (23, 64, 65) have been put forward as predictors of better upper limb function in children with unilateral CP.

However, whether or not ipsilateral “re-wiring” forms the basis for differential treatment responses remains a topic of debate (66). While some authors have suggested that children with unilateral CP with pure ipsilateral control are poor responders to intensive unimanual training (61, 67), others could not confirm these findings (68). These discrepancies reflect an on-going debate of how to determine the optimal therapy for the individual child with CP (48, 66). Additionally, the impact of the wiring pattern with respect to therapy outcome following different programs, i.e., bimanual training vs. intensive unimanual training, has not yet been systematically investigated.

The second premise is that if a child has a typical contralateral wiring pattern (i.e., the paretic hand is controlled via crossing fibers from the lesioned hemisphere only) or a bilateral wiring pattern (i.e., the affected hand is controlled by both hemispheres), the integrity of the lesioned CST_contra_ determines upper limb functional abilities. The structural integrity of descending motor pathways, based on, e.g., diffusion MRI measures of fractional anisotropy or fiber count of the CST_contra_ from the lesioned hemisphere to the affected hand, has been reported to predict good motor outcome (47, 69–73). We further hypothesize that those children with bilateral control of the affected hand and good structural integrity of the lesioned CST_contra_ will have ­better abilities to develop fine upper limb motor skills. Lastly, CST integrity has been reported to impact on treatment response, as demonstrated in children with acquired brain injuries. In this group of children, good structural integrity of the lesioned CST (measured at the level of the PLIC using DWI) was predictive for better functional gains following constraint-induced movement therapy (CIMT) (74). These results might be extrapolated to children with unilateral CP in case the affected hand is mainly controlled via the contralateral lesioned hemisphere.

Overall, the proposed categorization of children with unilateral CP based on their underlying CST wiring and structural integrity seems to be consistent with previous findings that demonstrated the link between clinical outcome measurements and (neuro)physiological, as well as brain MRI parameters at the group level (9, 26, 39). We specifically focus on the CST, given that it predominates skilled voluntary upper limb movements in humans and plays a crucial role in upper limb functional outcome as demonstrated in adult stroke (75, 76). However, inferring whether children exhibit predominantly ipsilateral vs. contralateral control of the affected hand is not easy and MM of the affected hand, as proposed in the current review, might only be a first indication of “ipsilateral or bilateral re-wiring” (26). Further research combining the systematic assessment of MM and TMS is an absolute necessity to further clarify the relationship between both measurements. Importantly, additional decision parameters with respect to upper limb functional outcome in children with unilateral CP might include (1) functional and structural connectivity patterns between sensory and motor areas, including the thalamocortical radiations (29, 40, 41); (2) functional and structural connectivity patterns between hemispheres (64); (3) underlying sensorimotor deficits, such as distal muscle weakness, spasticity, or impaired stereognosis (44); and (4) cognitive abilities and age (46). The importance of these measures and their integration into the proposed assessment to further optimize the categorization remain as future challenges. Lastly, given that the brain lesions occur while the nervous system is still developing, structural and functional connectivity may also be expected to change due to maturation and necessitate an age-corrected approach. If categorizing children with unilateral CP based on their CST wiring and structural integrity allows explaining the variability in treatment response, this may offer a real advantage with respect to individualized treatment planning and may even allow a stratified therapy approach in the future.

## Summary and Conclusion

Children with CP present with a striking heterogeneity in sensorimotor dysfunctions, which has triggered an increasing interest to optimize therapy in light of the specific requirements of the individual child, while keeping in mind that resources available for care and therapy are limited. Here, we advocate the idea that the individual “re-wiring” pattern and structural integrity of the CST might provide important information to further explain upper limb function and treatment outcome in children with unilateral CP and that CST functionality can be inferred from a systematic evaluation, which combines behavioral, neurophysiological, and medical imaging biomarkers. During the last decades, a large repertoire of neurophysiological and imaging techniques have been developed, but more research is required to identify which techniques are best suited to derive clinically relevant neural biomarkers. We provide a theoretical framework with a series of testable hypothesis for biomarker research and categorization in unilateral CP. Note that this framework has no immediate clinical application but presents a series of anatomically motivated ideas, which need to be tested and validated in future research in children with unilateral CP.

Lastly, we also indicate knowledge gaps regarding the availability of validated behavioral, neurophysiological, and medical imaging parameters, which can be used to further investigate the interaction between the CST wiring pattern, CST integrity, and upper limb sensorimotor outcome in unilateral CP. Characterizing whether the sensorimotor wiring pattern imposes a hard constraint on the maximum sensorimotor abilities a child can reach and/or on the response to treatment might pave the road for an evidence-based stratified therapy approach in CP.

## Conflict of Interest Statement

The authors declare that the research was conducted in the absence of any commercial or financial relationships that could be construed as a potential conflict of interest.
